# *Bridelia speciosa* Müll.Arg. Stem bark Extracts as a Potential Biomedicine: From Tropical Western Africa to the Pharmacy Shelf

**DOI:** 10.3390/antiox9020128

**Published:** 2020-02-02

**Authors:** Mohamad Fawzi Mahomoodally, Kouadio Ibrahime Sinan, Kouadio Bene, Gokhan Zengin, Giustino Orlando, Luigi Menghini, Serena Veschi, Annalisa Chiavaroli, Lucia Recinella, Luigi Brunetti, Sheila Leone, Paola Angelini, Vit Hubka, Stefano Covino, Roberto Venanzoni, Marie Carene Nancy Picot-Allain, Laura De Lellis, Alessandro Cama, Zoltán Cziáky, József Jekő, Claudio Ferrante

**Affiliations:** 1 Institute of Research and Development, Duy Tan University, Da Nang 550000, Vietnam or; 2 Department of Health Sciences, Faculty of Science, University of Mauritius, Réduit 230, Mauritius; picotcarene@yahoo.com; 3 Department of Biology, Science Faculty, Selcuk Universtiy, Campus, 42130 Konya, Turkey; sinankouadio@gmail.com; 4 Laboratoire de Botanique et Phytothérapie, Unité de Formation et de Recherche Sciences de la Nature, 02 BP 801 Abidjan 02, Université Nangui Abrogoua, 00225 Abidjan, Cote D’Ivoire; kouadio777@gmail.com; 5 Department of Pharmacy, “G. d’Annunzio” University Chieti-Pescara, 66100 Chieti, Italy; luigi.menghini@unich.it (L.M.); veschi@unich.it (S.V.); annalisa.chiavaroli@unich.it (A.C.); lucia.recinella@unich.it (L.R.); luigi.brunetti@unich.it (L.B.); sheila.leone@unich.it (S.L.); laura.delellis@unich.it (L.D.L.); alessandro.cama@unich.it (A.C.); claudio.ferrante@unich.it (C.F.); 6 Department of Chemistry, Biology and Biotechnology, University of Perugia, 06121 Perugia, Italy; paola.angelini@unipg.it (P.A.); stefano.covino@unipg.it (S.C.);; 7 Laboratory of Fungal Genetics and Metabolism, Institute of Microbiology of the Czech Academy of Sciences, 14220 Prague, Czech Republic; hubka@biomed.cas.cz; 8 Centre on Aging Sciences and Translational Medicine (Ce.S.I-Me.T), G. d’Annunzio University of Chieti-Pescara, 66100 Chieti, Italy; 9 Agricultural and Molecular Research and Service Institute, University of Nyíregyháza, 4400 Nyíregyháza, Hungary; cziaky.zoltan@nye.hu (Z.C.); jjozsi@gmail.com (J.J.)

**Keywords:** *Bridelia speciosa*, antioxidant, anti-proliferative, antimicrobial, phenolic compounds

## Abstract

*Bridelia* species have been used in traditional African medicine for the management of diverse human ailments. In the current work, the detailed phytochemical profiles of the extracts of the stem bark of *B. speciosa* were evaluated and the antioxidant and enzyme inhibitory properties of the extracts were assessed. The anti-bacterial and anti-mycotic effects of the extracts were evaluated against selected pathogen strains. Additionally, the anti-proliferative effects were studied on the liver cancer HepG2 cell line. Finally, the putative protective effects were assessed on isolated rat liver that was challenged with lipopolysaccharide (LPS). The results revealed the presence of 36 compounds in the ethyl acetate extract, 44 in the methanol extract, and 38 in the water extract. Overall, the methanol extract showed the highest antioxidant activity, particularly in LPS-stimulated rat liver. Additionally, this extract exerted the highest antimycotic effect on *C. albicans*, whereas the water extract showed a promising anti-proliferative effect on liver cancer HepG2 cells. The methanol extract was also the most active as enzyme inhibitor, against acetylcholinesterase and butyrylcholinesterase. The current study appraises the antioxidant and enzyme inhibition properties of *B. speciosa* methanol extract and showed that this specie could be a promising source of biologically active phytochemicals, with potential health uses.

## 1. Introduction

Plants have played a pivotal role in the progress of mankind, being considered as a substantial source of food and medicine. Traditionally used, for their curative properties among different populations of the world, medicinal plants are still considered to provide outstanding curative effects and they remain the most accessible therapeutic approach to a number of ailments. In traditional medicine, herbal remedies are prepared according to “standardized formula” transmitted from elders or shamans. Some of the preparation methods include decoction, infusion, maceration, tinctures, among others which can be administered by different routes, including optical, dermal, oral, nasal, and anal [1]. The WHO has publicized the need for documentation of ethnomedicinal data on plants, being conscious of the wealth of traditional knowledge that is related to medicinal use represents. Ethnomedicinal records make scientific validation easier and also provide rational regarding the use of plants/herbal preparations for the management of specific ailments [2]. Global public interest for plants-derived products has undoubtedly increased and, today, one of the challenges is to provide scientific evidences of claimed biological activity, but also to unlock the potential of underexplored medicinal plants.

Recently, several endeavors have been made to probe for new sources of bioactive compounds from natural raw materials [3,4]. Among them, the bark of plants is one of the most important sources of bioactive compounds, including phenolics, flavonoids, and terpenes. In addition, extracts that were prepared from barks have been reported to possess broad biological activities, such as antioxidant, antimicrobial, or anti-cancer [3,4,5,6,7]. Based on these data, new studies on uninvestigated bark samples, particularly from Africa, might lead to the discovery of novel bioactive compounds for potential uses in the nutraceutical and pharmaceutical industries.

The *Bridelia* genus consists of approximately 60–70 species distributed in tropical and subtropical regions of the globe, particularly in Asia and Africa [8]. Several species of this genus have been used in traditional medicinal systems for the management of multiple diseases, including diabetes, urinary stones, lumbago, rheumatism, venereal diseases, bronchitis, gastrointestinal problems, cardiac pain, infertility, epilepsy, and diarrhoea, among others [9]. Keeping this in view, the biological efficacy of several *Bridelia* species has been claimed in several research pieces [10,11,12,13,14,15]. In earlier studies, the chemical profiles of the members of the *Bridelia* genus have been reported. For example, previous studies have reported the presence of phenolic acids (gallic acid and ellagic acid, etc.), tannins, and flavonoids in several *Bridelia* species, including *B. ferruginea, B. micrhanta,* and *B. retusa.* Such studies also highlighted the importance of the *Bridelia* genus, which could open avenues for new studies [16,17,18].

As far as our literature search could ascertain, little scientific information was available on *B. speciosa*. In this perspective, the current work aims at characterizing the stem bark extracts of *B. speciosa* investigating phytocompounds and elucidating the antioxidant, enzyme inhibitory properties, protective and anti-proliferative effects in experimental models of liver cancer and inflammation.

## 2. Materials and Methods

### 2.1. Plant Material and Preparation of Extracts

The plant samples were collected from wild areas in Gontougo region (Nioumassi) of Ivory Coast in 2018 and they were identified by Dr. Kouadio Bene, botanist at the Laboratoire de Botanique et Phytothérapie, Université Nangui Abrogoua, Abidjan, Côte d’Ivoire. A voucher specimen of the plant material was deposited at Selcuk University, Science Faculty (KIS-1005). The stem barks samples were randomly collected from ten plants in a same population. The stem barks samples were taken stripped vertically while using a knife to the limit of the cambium layer. The stem barks were separated and then dried at room temperature for ten days.

One laboratory mill (Retsch Cutting Mill SM 200, Haan, Germany) was used to powder them (about 2 mm). The extraction procedure was conducted following traditional maceration (for ethyl acetate and methanol) and infusion (for water) methods. Briefly, for maceration, 5 g powdered plant samples was stirred with solvents (100 mL) overnight at the room temperature. Subsequently, the solvents were evaporated using a rotary-evaporator. For water extracts, 5 g powdered plants in boiled water (100 mL) was allowed to stand for 20 min. The aqueous extract was then lyophilized and all of the extracts were kept in +4 °C until use.

### 2.2. Chemicals

The chemicals were purchased from Sigma–Aldrich (Darmstadt, Germany). They were: 2,2’-azino-bis(3-ethylbenzothiazoline-6-sulphonic acid (ABTS), 1,1-diphenyl-2-picrylhydrazyl (DPPH), gallic acid, rutin, caffeic acid, electric eel acetylcholinesterase (AChE) (type-VI-S, EC 3.1.1.7), horse serum butyrylcholinesterase (BChE) (EC 3.1.1.8), galantamine, acetylthiocholine iodide (ATChI), butyrylthiocholine chloride (BTChI) 5,5-dithio-bis(2-nitrobenzoic) acid (DTNB), tyrosinase (EC1.14.18.1, mushroom), glucosidase (EC. 3.2.1.20, from *Saccharomyces cerevisiae*), amylase (EC. 3.2.1.1, from porcine pancreas), sodium molybdate, sodium nitrate, sodium carbonate, Folin-Ciocalteu reagent, hydrochloric acid, sodium hydroxide, trolox, EDTA, neocuproine, cupric chloride, ammonium acetate, ferric chloride, 2,4,6-Tris(2-pyridyl)-s-triazine (TPTZ), ammonium molybdate, ferrozine, ferrous sulphate hexahydrate, kojic acid and acarbose. All of the chemicals were of analytical grade.

### 2.3. Phytochemical Composition

The total bioactive compounds were determined colorimetrically, as described previously [19,20,21]. The results were expressed as mg of standard compounds (gallic acid for phenolic; and rutin for flavonoids; caffeic acid for total phenolic acid; catechin for total flavanol and tannins; quillaja for saponins) per g of dried extract. The bioactive profile of the *B. speciosa* extracts was determined while using a Dionex Ultimate 3000RS UHPLC instrument. All of the analytical and chromatographic details are given in Supplemental Materials. *B. speciosa* water and methanol extracts (5 µg/mL) were also analyzed for accurate phenolic quantitative determination of epicatechin, catechin, and gallic acid while using a reversed phase HPLC-fluorimetric in gradient elution mode, as recently described [22]. The experimental details are given in Supplemental Materials.

### 2.4. Determination of Antioxidant and Enzyme Inhibitory Effects

For antioxidant capacity, different test systems, including radical quenching, reducing power, phosphomolybdenum, and ferrous ion chelating, were used. The methods details were described in our earlier paper [23]. Standard trolox and EDTA equivalents were selected as standards to explain results. For enzyme inhibitory effects, key enzymes for global health problems were selected, namely α-amylase and α-glucosidase, acetylcholinesterase (AChE), butyrylcholinesterase (BChE), and tyrosinase. Similar to the antioxidant assays, standard equivalent method (acarbose for amylase and glucosidase; galatamine for AChE and BChE; kojic acid for tyrosinase) was selected [23]. Experimental details are given in the Supplemental Materials.

### 2.5. Antimicrobial and Antimycotic Assays

Antibiotic and antimycotic assays were performed according to our previous studies [24,25]. The detailed description is reported in the Supplementary Material.

### 2.6. Cell lines and Treatments

Human hepatocellular carcinoma HepG2 cells were cultured in DMEM (Sigma, St. Louis, MO, USA) that was supplemented with 10% FBS at 37 °C, 5% CO_2_. *B. speciosa* methanol and water extracts were solubilized in phosphate-buffered saline (PBS) by sonication and then centrifuged at 1200 rpm for five minutes to remove the insoluble fraction. Supernatants were filtered through 0.2 μm pore diameter filters (Euroclone).

### 2.7. Cell Viability Assay

Cell viability was tested by MTT assay [3-(4,5-Dimethyl-2-thiazolyl)-2,5-diphenyl-2H-tetrazolium bromide; Sigma, St. Louis, MO, USA]. Briefly, the HepG2 cells were seeded in 96-well plates (5 × 103 cells/well) and they were treated the following day for 24, 48, or 72 hours with *B. speciosa* methanol and water extracts at various concentrations as indicated, or with vehicle (control). The MTT assay was performed as previously described [26]. The IC_50_ values were calculated while using the CompuSyn software.

### 2.8. Ex Vivo Studies

Sprague–Dawley rats (200–250 g) were sacrificed by CO_2_ inhalation (100% CO_2_ at a flow rate of 20% of the chamber volume per min) and the liver specimens were immediately collected and then maintained at 37 °C for 4 h in RPMI that was supplemented with *E. coli* LPS (10 µg/mL). During the incubation period, liver specimens were treated with either the methanol and water extracts of *B. speciosa* (10–500 µg/mL). Afterwards, the tissues were homogenized in 50 mM perchloric acid solution for biochemical determinations, as following described. The liver dopamine (DA) and 3-hydroxy-kinurenine (3-HK) levels were analyzed through an HPLC apparatus (Jasco PU 2080-plus) coupled to electrochemical detection (ESA Coulochem III). All of the details were given in our earlier paper [27].

### 2.9. Statistical Analysis

The results are given as mean ± S.D. One-way analysis of variance (ANOVA) with post-hoc Tukey test was conducted to determine significant differences (in total bioactive compounds, antioxidant, and enzyme inhibitory assays) between the extracts. ANOVA coupled to Newman–Keuls post-hoc test was employed for statistical analysis of data in pharmacological in vitro and ex vivo assays. GraphPad Prism 5.01 software was used to perform all of the statistical analyses. The results were considered to be statistically significant at *p* < 0.05.

## 3. Results and Discussion

While the term “phenolic compounds” includes a broad group of molecules containing at least one phenol unit, different other subgroups, such as flavonoids, tannins, and phenolic acids, among others, can be defined based on their chemical structures [28]. In the current work, the content of major phytochemical groups from *B. speciosa* stem bark extracts was evaluated while using spectrophotometric methods and are summarized in Table 1. The best concentration of flavanols, tannins, and saponins was observed in the *B. speciosa* stem bark methanol extract, whereas the highest flavonoid content was obtained in ethyl acetate extract. Flavonoids encompass a group of secondary metabolites having a distinct polyphenolic structure that consists of 15-carbon skeleton (two phenyl rings and one heterocyclic ring) [29]. On the other hand, flavanols or flavan-3-ols, which represent a popular group of flavonoids, include epicatechin and catechin, and their polymerization products [30]. Phenolic acids, which are the most widely distributed plant non-flavonoid phenolic compounds [31], carboxylic acids derivatives of either benzoic or cinnamic acid skeletons [32], were not identified from the ethyl acetate extract. However, HPLC-MS/MS was employed to more accurately evaluate the phytocomposition of *B. speciosa* stem bark extracts while considering the limitations of spectrophotometric determination in the assessment of phytocompound composition of herbal extract. With regards to the different extracts, 36 compounds were identified from the ethyl acetate extract, 44 from the methanol and 38 from the water extract of *B. speciosa* stem bark (Table 2). Ellagic, quinic, shikimic, gallic, and ferulic acids were characterized in all of the extracts, albeit the spectrophotometric determination did not reveal the presence of phenolic acids. Corilagin, an ellagitannin, having [M − H]^−^ at *m/z* 633 was also characterized from all of the *B. speciosa* extracts. Mallotinic acid or its isomer, a hydrolyzable tannin [32] having [M − H]^−^ at *m/z* 801, was identified from the methanol and water extracts. Bruguierol A ([M + H]^+^ at *m/z* 191), a dammarane triterpene [33] and Prodelphinidin B ([M − H]^−^ at *m/z* 609), a polymeric tannin composed of gallocatechin, were characterized from the ethyl acetate and methanol extracts of *B. speciosa* stem bark. Tryptamine, which is a monoamine alkaloid, having [M − H]^−^ at *m/z* 161, was identified from the methanol extract of *B. speciosa* stem bark only. Four unidentified tannins were identified from *B. speciosa* stem bark methanol extract.

In the current work, radical scavenging, reducing power, and metal chelating assays were used to assess the antioxidant properties of *B. speciosa* stem bark extracts. This approach is believed to provide more accurate and comprehensive information regarding the antioxidant potential of herbal extracts [34]. The methanol extract was the most active in phosphomolybdenum assay, followed by the water and the ethyl acetate extracts (Table 3). A similar order was also obtained for total phenolic content (methanol extract >water extract >ethyl acetate extract). A positive correlation between concentration of phenols and antioxidant capacity was claimed in other studies, which suggests that high phenolic content could be an index of antioxidant capacity [35,36]. Deleterious effects of free radicals that are mainly caused by their instability and high reactivity lead to lipid, protein, and DNA alterations, thereby triggering diseases [37]. In line with the phosphomolybdenum assay, the methanol extract of *B. speciosa* stem bark was the most effective scavenger of DPPH (495.45 mg TE/g extract) and ABTS (902.33 mg TE/g extract). Apart from radical scavenging, the electron-donating capacity, as measured in terms of reducing power, is also regarded as an important antioxidant mechanism [38]. The CUPRAC and FRAP are the most common methods for measuring reducing power, in vitro. The FRAP evaluates the ability of the herbal extract to reduce ferric to ferrous, whereas the CUPRAC assay measures the conversion of cupric to cuprous [39]. Likewise, *B. speciosa* stem bark methanol extract showed the highest reducing activity (1325.89 and 952.68 mg TE/g extract, for CUPRAC and FRAP, respectively). However, the methanol extract (12.98 mg EDTAE/g) was the least active metal chelator. Indeed, transition metals, such as iron, can participate in Fenton reaction, converting hydrogen peroxide that is produced from mitochondrial oxidative respiration, into highly toxic hydroxyl free radical [40]. In the current study, the ethyl acetate extract, rich in flavonoids, showed the highest metal chelating properties (Table 3).

Most of the therapeutic drugs that are clinically available function by inhibiting a specific enzyme [41]. Today, the challenge is to find novel inhibitors that can effectively correct metabolic imbalances, without causing side effects. The ability of *B. speciosa* stem bark extracts to inhibit cholinesterases, tyrosinase, α-amylase, and α-glucosidase was assessed and is reported in Table 4. The inhibition of cholinesterases, namely acetylcholinesterase and butyrylcholinesterase, remains the main focus for the management of Alzheimer’s disease. Besides, an increasing number of publications and clinical studies substantiates that Alzheimer’s disease and type 2 diabetes might share some pathophysiological similarities [42,43]. As such, type 2 diabetes has been identified as a risk factor for Alzheimer’s disease based on multiple connections [44]. From Table 4, it was noted that *B. speciosa* stem bark methanol extract exhibited the highest inhibition against acetylcholinesterase (4.98 mg GALAE/g extract) and butyrylcholinesterase (5.14 mg GALAE/g extract). The extracts showed a relatively low inhibition against α-amylase (ranging from 0.59 to 1.20 mmol ACAE/g extract), whereas only the ethyl acetate extract (3.56 mmol ACAE/g extract) actively inhibited α-glucosidase. Tyrosinase inhibition is the main therapeutic strategy for the management of epidermal hyperpigmentation conditions. The methanol extract (157.25 mg KAE/g extract) of *B. speciosa* stem bark showed highest inhibitory activity against tyrosinase. The observed enzyme inhibitory activity of *B. speciosa* stem bark methanol extract might be related to more than one phytochemical that is present in the extracts. Interestingly, molecular docking studies have previously shown that mangiferin effectively binds to acetylcholinesterase and butyrylcholinesterase [45]. Ferulic acid was reported to hybridise with quinoline in a competitive manner. Carbazole was reported to be more potent than galantamine, which showed pronounced inhibition against cholinesterases [46,47]. On the other hand, it was also reported that corilagin (IC_50_ = 1.231 mM) was active against tyrosinase [48]. Mangiferin was reported to exert a non-competitive inhibition of α-glucosidase [49].

**Figure 1:**
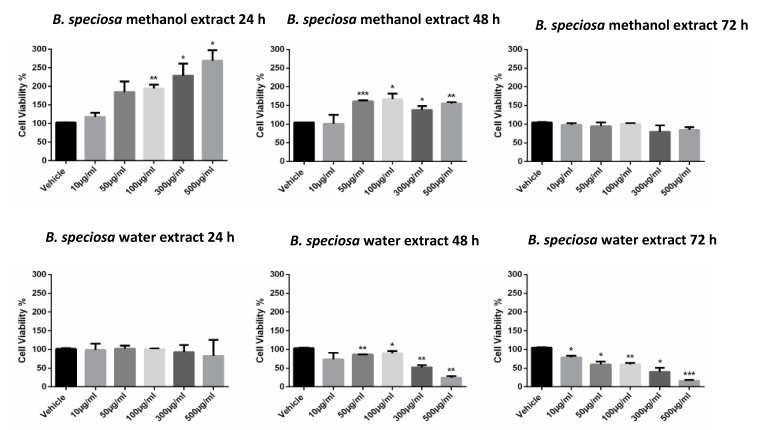
*B. speciosa* methanol and water extracts affect cell viability in human hepatocellular carcinoma HepG2. Cell viability was assessed by MTT assay after incubation for 24, 48, or 72 h, with the extracts at various concentrations as indicated, or with vehicle (control). Data shown are the means + SD of two independent experiments with quadruplicate determinations. Statistical analyses were performed using GraphPad Prism version 5.01 software (San Diego, CA). Comparisons of mean values between control and each drug concentration were performed by an unpaired Student’s t-test. A *p*-value ≤ 0.05 was considered statistically significant (* *p* < 0.05; ** *p* < 0.01; *** *p* < 0.001).

**Figure 2:**
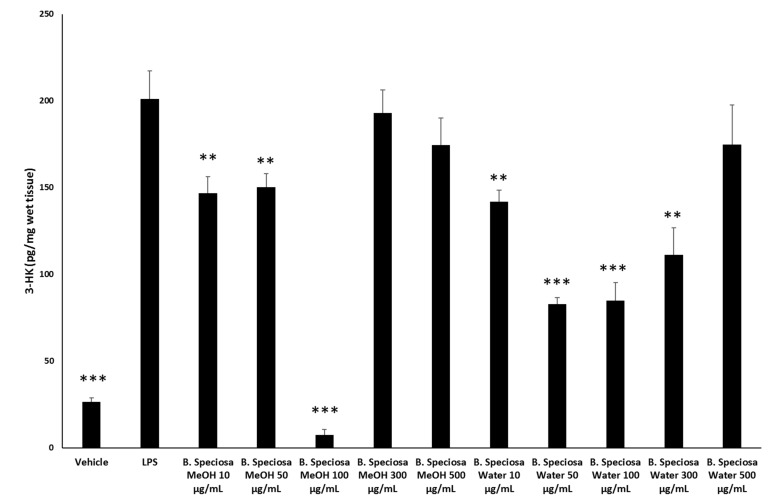
Effects of *B. speciosa* methanol and water extracts on LPS-induced 3-HK level in isolated rat liver specimens. ANOVA, *p* < 0.0001; ** *p* < 0.01, *** p < 0.001 vs. LPS control group.

**Figure 3:**
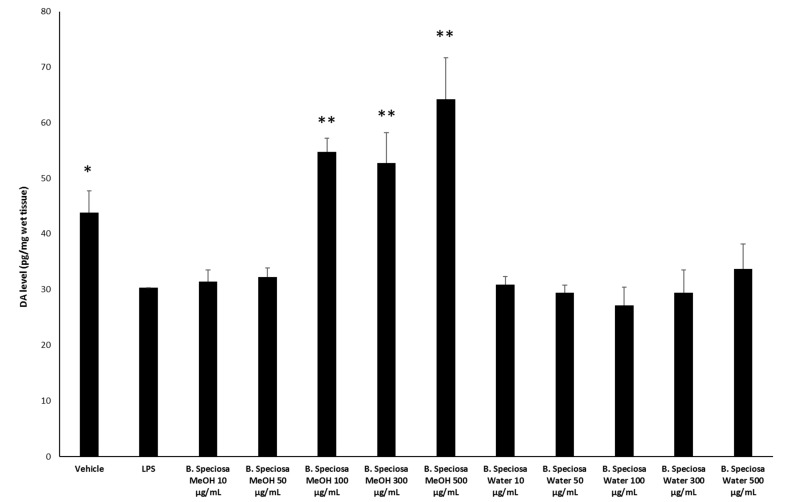
Effects of *B. speciosa* methanol and water extracts on LPS-induced DA level in isolated rat liver specimens. ANOVA, *p* < 0.001; * *p* < 0.05, ** p < 0.01 vs. LPS control group.

The methanol and water extracts were selected for further biological assays based on the results of colorimetric analyses indicating a more promising phytochemical profile in terms of total phenols and antiradical activity. A microbiological study was carried out to investigate the potential anti-fungal and anti-bacterial effects of selected pathogen strains, which are fully described in Supplementary Materials. The anti-microbial effects of both water and methanol extracts were compared with reference drugs and presented in Table 5 and Table 6. The results clearly demonstrated that the extracts were less effective when compared to the reference drugs, namely the anti-mycotics fluconazole and griseofulvin and the anti-bacterial ciprofloxacin. Nevertheless, the methanol extract of *B. speciosa* displayed anti-mycotic activity on *C. albicans* (YEPGA 6379) that deserves further investigation. This inhibitory effect is consistent with its major content in total phenolic compounds [24,25], as confirmed by both colorimetric assays (Table 1) and independent HPLC-fluorimetric analysis (Table 7). While considering both the incidence of *C. albicans* opportunistic infections occurring in liver disorders [50] and the traditional use of *Bridelia* genus [9], a pharmacological investigation was subsequently performed to explore anti-proliferative effects against the liver cancer HepG2 cell line and protective effects on isolated rat liver specimens challenged with the LPS pro-inflammatory stimulus. The HepG2 cell viability was evaluated through the MTT test, which revealed a stimulatory effect induced by methanol extract, up to 48 hours following treatment (Figure 1). Conversely, the water extract of *B. speciosa* was able to reduce cell viability in the concentration range (300–500 µg/mL) throughout the 72 hour treatment (Figure 1), thus indicating significant anti-proliferative effects that are consistent with more than one speculation. On one side, the concentration-dependent anti-proliferative effects that were exerted by the water extract could be related to epicatechins [51]. This was, at least in part, confirmed by the mild anti-proliferative effect that was exerted by the sole epicatechin (100 µg/mL) (CTR: 100; Epicatechin: 87.88 ± 4.35). On the other hand, we cannot exclude that the stimulating effect induced by methanol extract depends on its major content in phenolic compounds (Table 1 and Table 7), with particular regards to gallic acid, which could also exert putative pro-oxidant effects [22,52], thus potentially contributing to the maintenance of a microenvironment favorable to the proliferation of a cancer cell line [24]. Protective effects following extract treatment were also evaluated in a toxicological model constituted by isolated liver specimens that were stimulated with LPS, which increased the 3-HK levels in the liver tissue (Figure 2). 3-HK is a kinurenine-3-monooxygenase (KMO)-deriving kinurenine metabolite that is able to induce oxidative stress in multiple tissues, including the brain and pancreas [53,54]. Additionally, increased KMO activity was described in rodent models of acute pancreatitis [53], despite that there is still a lack of scientific evidence about KMO activity and 3-HK level in inflamed liver. Conversely, liver dopamine (DA) levels were reduced in the same experimental condition (Figure 3). Besides its crucial role as neurotransmitter in the central nervous system, recent findings suggest anti-inflammatory and protective effects that are induced by DA administration in experimental models of acute pancreatitis and hepatitis [55,56]. Consistent with the observed antiradical activity, both of the extracts reduced the LPS-induced levels of 3-HK (Figure 2). On the other hand, the water extract was completely ineffective against the LPS-induced levels of DA, thus ruling out the involvement of DA in mediating extract anti-oxidant effects, in the liver. By contrast, the methanol extract displayed a significant stimulating effect on liver DA concentration (Figure 3). This protective effect is consistent with both the antioxidant effect that is exerted by the extract and with literature [22,27]. Overall, the present pharmacological assays suggest that *B. speciosa* could be considered as a source of natural compounds with potential application in liver inflammation and cancer.

## 4. Conclusions

This is the first report regarding the biological and phytochemical profiles of *B. speciosa* stem bark extracts. In this respect, our findings can be considered as the first attempt to provide new scientific information on the *Bridelia* genus. Among the three extracts studied, the methanol extract showed antioxidant and inhibitory properties against enzymes that are related to Alzheimer’s disease and epidermal hyperpigmentation conditions. The antioxidant effects displayed by the methanol extract were also consistent with the observed protective effects in the liver and the anti-mycotic effect against the *C. albicans* (YEPGA 6379) strain. The protective effects on rat liver induced by methanol extract were also substantiated by the increased DA and reduced 3-HK levels. On the other hand, the water extract reduced the HepG2 cell viability, thus suggesting potential anti-proliferative effects. Several compounds identified and quantified in the *B. speciosa* stem bark water and methanol extracts, including gallic acid and catechins, might be responsible for the observed effects. Therefore, the isolation of compounds from the methanol and water extracts is required for the validation of the observed pharmacological investigations. To sum up, our findings suggest that *B. speciosa* barks may be a key bio-resource for the development of novel pharmaceuticals or cosmeceuticals. Further studies are strongly recommended for exploring more biological properties through in vivo animal studies.

## Figures and Tables

**Table 1:** Total bioactive components of the tested samples.

Samples	Total phenolic Content (mg GAE/g Extract)	Total Flavonoid Content (mg RE/g Extract)	Total Phenolic Acid Content (mg CAE/g)	Total Flavanol Content (mg CE/g)	Total Tannin Content (mg CE/g)	Total Saponin Content (mg QE/g)
EA	38.42 ± 0.38 ^c^	5.85 ± 0.12 ^a^	nd	3.61 ± 0.02^c^	3.28 ± 0.38 ^c^	177.82 ± 14.15 ^c^
MeOH	224.28 ± 1.08 ^a^	1.51 ± 0.04 ^b^	11.55 ± 1.31 ^b^	246.28 ± 10.63 ^a^	324.09 ± 10.99 ^a^	1031.45 ± 48.83 ^a^
Water	210.29 ± 0.71 ^b^	1.44 ± 0.17 ^b^	13.91 ± 0.42 ^a^	6.15 ± 0.18 ^b^	67.83 ± 3.64 ^b^	772.56 ± 56.39 ^b^

Values expressed are means ± S.D. of three parallel measurements. GAE: Gallic acid equivalent; RE: Rutin equivalent; CE: catechin equivalent; CAE: caffeic acid equivalent; QE: Quillaja equivalent; EA: Ethyl acetate; MeOH: Methanol; nd: not detected. Different letters indicate significant differences in the extracts (*p* < 0.05).

**Table 2:** Chemical composition of the tested extracts.

No.	Name	Class ^3^	Formula	Rt ± 0.03 min	[M + H]^+^	[M − H]^−^	Fragment 1	Fragment 2	Fragment 3	Fragment 4	Fragment 5	Detected in Extract ^2^
1	Quinic acid	a	C7H12O6	1.23		191.05557	173.0447	127.0388	111.0438	93.0331	85.0280	A,B,C
2	Shikimic acid	a	C7H10O5	1.31		173.04500	155.0338	137.0234	111.0439	93.0331	73.0280	A,B,C
3	Citric acid	a	C6H8O7	1.57		191.01918	173.0082	129.0181	111.0074	87.0073	85.0280	B,C
4	Prodelphinidin B	b	C30H26O14	1.73		609.12444	441.083	423.073	305.0672	177.0185	125.0231	B,C
5 ^1^	Gallic acid (3,4,5-Trihydroxybenzoic acid)	c	C7H6O5	2.29		169.0137	125.0231	97.0282	81.0332	79.0175	69.0329	A,B,C
6	Gallocatechin (Casuarin, Gallocatechol)	d	C15H14O7	4.52		305.06613	261.0767	219.0651	167.0341	137.0234	125.0232	A,B,C
7 ^1^	Tryptamine	e	C10H12N2	8.44	161.107875		144.0810	143.0732	117.0703	115.0546	103.0547	B,C
8	Syringic acid-4-O-glucoside	f	C15H20O10	10.57		359.09783	197.0451	182.0214	153.0546	138.031	123.0073	C
9 ^1^	Catechin (Catechol, Catechuic acid)	d	C15H14O6	13.28		289.07121	245.082	203.0711	151.0389	125.0233	109.028	B,C
10 ^1^	Epigallocatechin (Epigallocatechol)	d	C15H14O7	13.57		305.06613	261.0767	219.0658	167.0339	137.0234	125.0232	A,B,C
11 ^1^	Vanillin (4-Hydroxy-3-methoxybenzaldehyde)	g	C8H8O3	15.47	153.05517		125.0601	111.0445	110.0367	93.0341	65.0393	A,B,C
12 ^1^	Epigallocatechin-3-O-gallate (Teatannin II)	d	C22H18O11	16.39		457.07709	305.0661	169.0131	161.0238	125.0231		A,B,C
13 ^1^	Gallocatechin-3-O-gallate	d	C22H18O11	16.40		457.07709	305.067	169.0133	161.0233	125.0231		C
14	Dihydrokaempferol-O-hexoside	d	C21H22O11	17.02		449.10839	287.0568	269.0447	259.0607	125.023		A,B,C
15 ^1^	Epicatechin	d	C15H14O6	17.04		289.07121	245.0818	203.0706	151.0388	125.0231	109.028	B,C
16	3,5-Dimethoxy-4-hydroxybenzaldehyde (Syringaldehyde)	g	C9H10O4	17.24	183.06574		155.0705	140.0469	123.0444	105.0341	95.0498	A,B
17	Corilagin	h	C27H22O18	17.49		633.07279	463.0526	419.0627	300.9995	275.0205	169.0134	A,B,C
18	Mangiferin (Aphloiol, Chinonin)	i	C19H18O11	18.41		421.07709	343.0459	331.0464	301.0358	272.033	259.0249	A,B,C
19	Unidentified tannin 1	h	C34H26O22	18.96		785.08375	633.0741	300.9992	275.0205	125.0229		B
20	Ferulic acid	c	C10H10O4	19.25		193.05009	178.0259	149.0594	137.023	134.0364	121.028	A,B,C
21	Mallotinic acid or isomer	h	C34H26O23	19.28		801.07867	757.0872	633.0753	613.047	463.0517	300.9995	B,C
22 ^1^	Epicatechin-3-O-gallate	d	C22H18O10	19.37		441.08218	289.0725	271.0614	245.0808	169.0132	125.023	B,C
23	Loliolide	j	C11H16O3	19.47	197.11777		179.1071	161.0963	135.1172	133.1016	107.0861	A,B,C
24	Unidentified tannin 2	h	C41H30O27	19.63		953.08963	300.9994	275.02	249.0387			B,C
25	Ellagic acid-4-O-glucoside	k	C20H16O13	19.90		463.05127	300.9995	299.9915				A,B,C
26	4-Hydroxy-3-methoxycinnamaldehyde (Coniferyl aldehyde)	g	C10H10O3	19.97	179.07082		161.0599	147.0443	133.0654	119.0496	55.0187	A,B,C
27	Unidentified tannin 3	h	C34H26O22	20.08		785.08375	633.0734	300.9994	275.0207			B
28	Isoferulic acid	c	C10H10O4	20.30		193.05009	178.0264	149.06	137.0232	134.0362	121.0283	A
29	Unidentified tannin 4	h	C34H26O22	21.25		785.08375	300.9996	275.0205	249.0402	125.0228		B,C
30	Myricitrin (Myricetin-3-O-rhamnoside)	d	C21H20O12	21.96		463.08765	317.0292	316.023	287.0213	271.0255	178.9978	B,C
31	Di-O-methylellagic acid-O-hexoside	k	C22H20O13	22.16		491.08257	476.0599	328.023	312.9996	297.9761		A,B,C
32	Ellagic acid-O-pentoside	k	C19H14O12	22.76		433.04071	300.9994	299.9916	283.9974	257.0082		A,B,C
33	Eschweilenol C (Ellagic acid-4-O-rhamnoside)	k	C20H16O12	23.09		447.05636	300.9994	299.9916				A,B,C
34	Pentahydroxyflavone-C-hexoside	d	C21H20O12	23.11	465.10331		447.0935	429.0806	369.0611	327.0503	303.0504	A
35	Ellagic acid	k	C14H6O8	23.38		300.99845	283.9967	257.0094	229.0138	201.0187	185.0237	A,B,C
36	Dimethoxy-trihydroxyflavone-O-hexoside	d	C23H24O12	24.29		491.11895	328.0586	313.0352	299.0195	285.0397	271.0252	B,C
37	Di-O-methylflavellagic acid O-hexoside	k	C21H18O13	24.70		507.07749	344.0187	328.994	313.97			A
38	Ducheside A (3-O-Methylellagic acid-4′-O-xyloside)	k	C20H16O12	24.74		447.05636	315.0151	314.0074	299.9917	298.983	270.9886	A,B,C
39	3,3′-Di-O-methylellagic acid-O-pentoside	k	C21H18O12	25.32		461.07201	446.0498	328.0228	312.9995	297.9757		A,B,C
40	3,3′,4-Tri-O-methylflavellagic acid-4-O-glucoside	k	C23H22O14	25.55		521.09314	506.0705	491.0473	358.0327	343.0098	327.9864	A,B,C
41	Eschweilenol A or isomer	k	C20H10O11	25.90		425.01449	300.9993	299.9917	298.9837			B
42	Dihydroactinidiolide	j	C11H16O2	26.58	181.12286		163.112	145.1015	135.1172	121.1015	107.0861	A,B,C
43	Di-O-methylellagic acid acetylhexoside	k	C24H22O14	27.49		533.09313	328.0231	312.9999	297.9756	269.9827		A
44	3,3′-Di-O-methylellagic acid	k	C16H10O8	27.84		329.02975	314.0073	298.9837	270.9887			A,B,C
45	Sebacic acid	a	C10H18O4	27.96		201.11268	183.102	157.1229	139.1117	111.0801		A
46	3,3′,4-Tri-O-methylellagic acid	k	C17H12O8	30.18		343.0454	328.0231	312.9995	297.9758	285.0038		A
47	Undecanedioic acid	a	C11H20O4	30.85		215.12834	153.1273	125.0956				A
48	3,3′,4-Tri-O-methylflavellagic acid	k	C17H12O9	31.21		359.04031	344.0171	328.9948	313.9717	300.9995		A,B,C
49	3,3′,4,4′-Tetra-O-methylellagic acid	k	C18H14O8	32.00	359.0767		344.0533	343.0448	329.0295	313.0347		A,B,C
50	Dihydroxy-trimethoxyflavone	d	C18H16O7	33.10		343.08178	328.0585	313.0359	298.0118			B
51	Bruguierol A	l	C12H14O2	36.06	191.10721		173.0965	161.0966	147.0801	135.0807	107.0496	A,B
52^1^	Linoleic acid	a	C18H32O2	45.69		279.23241	261.2231	59.0124				A,B
53	Pheophytin A	m	C55H74N4O5	62.94	871.57375		593.277	533.2559	460.2264			A,B

^1^ Confirmed by standard. ^2^ A: Ethyl acetate extract; B: Methanol extract; C: water extract. ^3^ a: carboxylic acid; b: polyflavonoid; c: phenolic acid; d: flavonoid; e: alkaloid; f: phenolic acid glucoside; g: phenolic aldehyde; h: tannin; i: xanthon; j: benzofuran; k: benzopyrane; l: phenolic heterocycle; m: porphyrin.

**Table 3:** Antioxidant activities of the tested samples.

Samples	Phosphomolybdenum (mmol TE/g)	DPPH (mg TE/g Extract)	ABTS (mg TE/g Extract)	CUPRAC (mg TE/g Extract)	FRAP (mg TE/g Extract)	Metal Chelating Ability (mg EDTAE/g)
EA	2.24 ± 0.07 ^c^	18.62 ± 0.39 ^c^	14.82 ± 0.45 ^c^	94.34 ± 0.82 ^c^	46.13 ± 0.58 ^c^	32.08 ± 1.60 ^a^
MeOH	5.89 ± 0.37 ^a^	495.45 ± 0.53 ^a^	902.33 ± 2.41 ^a^	1325.89 ± 30.05 ^a^	952.68 ± 23.61 ^a^	12.98 ± 0.10 ^b^
Water	5.17 ± 0.14 ^b^	463.86 ± 14.04 ^b^	581.14 ± 33.94 ^b^	1082.42 ± 3.72 ^b^	850.05 ± 5.35 ^b^	14.28 ± 2.15 ^b^

Values expressed are means ± S.D. of three parallel measurements. TE: Trolox equivalent; EDTAE: EDTA equivalent; EA: Ethyl acetate; MeOH: Methanol. Different letters indicate significant differences in the extracts (*p* < 0.05).

**Table 4:** Enzyme inhibitory properties of the tested extracts.

Samples	AChE (mg GALAE/g Extract)	BChE (mg GALAE/g Extract)	Tyrosinase (mg KAE/g Extract)	α-Amylase (mmol ACAE/g Extract)	α-Glucosidase (mmol ACAE/g Extract)
EA	4.56 ± 0.20 ^b^	3.59 ± 0.05 ^b^	119.80 ± 1.30 ^c^	0.86 ± 0.03 ^b^	3.56 ± 0.03
MeOH	4.98 ± 0.04 ^a^	5.14 ± 0.08 ^a^	157.25 ± 0.48 ^a^	1.20 ± 0.01 ^a^	na
Water	3.60 ± 0.15 ^c^	2.61 ± 0.31 ^c^	137.49 ± 0.35 ^b^	0.59 ± 0.04 ^c^	na

Values expressed are means ± S.D. of three parallel measurements. AChE: acetylcholinesterase; BChE: butyrylcholinesterase; GALAE: Galantamine equivalent; KAE: Kojic acid equivalent; ACAE: Acarbose equivalent; na: not active; EA: Ethyl acetate; MeOH: Methanol. Different letters indicate significant differences in the extracts (*p* < 0.05).

**Table 5:** Minimal inhibitory concentrations (MICs) of *B. speciosa* water and methanol extracts, fluconazole, and griseofulvin against clinical yeasts and dermatophytes.

	MIC (µg mL^−1^) *			
Fungal Strains	Methanol Extract	Water Extract	Fluconazole	Griseofulvin
*Candida albicans* (YEPGA 6183)	396.85 (250–500)	198.42 (125–250)	2	>8
*Candida albicans* (YEPGA 6379)	49.6 (31.25–62.5)	78.74 (62.5–125)	1	>8
*Candida tropicalis* (YEPGA 6184)	629.96 (500–1000)	396.85 (250–500)	4	>8
*Candida parapsilosis* (YEPGA 6551)	78.74 (62.5–125)	99.21 (62.5–125)	2	>8
*Arthroderma crocatum* (IHEM 5251)	157.49 (125–250)	78.74 (62.5–125)	8	>8
*Arthroderma crocatum* (CCF 5207)	99.21 (62.5–125)	78.74 (62.5–125)	>16	>8
*Arthroderma insingulare* (CCF 5417)	157.49 (125–250)	39.37 (31.25–62.5)	>16	>8
*Arthroderma quadrifidum* (CCF 5792)	198.42 (125–250)	78.74 (62.5–125)	>16	>8
*Trichophyton erinacei* (CCF 5930)	314.98 (250–500)	157.49 (125–250)	>16	0.25
*Trichophyton interdigitale* (CCF 4823)	99.21 (62.5–125)	49.61 (31.25–62.5)	>16	1
*Trichophyton rubrum* (CCF 4879)	78.74 (62.5–125)	78.74 (62.5–125)	8	2
*Trichophyton tonsurans* (CCF 4834)	157.49 (125–250)	39.58 (31.25–62.5)	2	0.125

* MIC values are reported as geometric means of three independent replicates (*n* = 3); MIC range concentrations are reported within brachets. CCF, Culture Collection of Fungi, Department of Botany, Charles University, Prague, Czech Republic; IHEM, Belgian Coordinated Collections of Micro-organisms (BCCM/IHEM), Brussels, Belgium; YEPGA, yeast extract-peptone-glucose agar.

**Table 6:** Minimum inhibitory concentration (MIC) of *B. speciosa* extracts and ciprofloxacin towards selected bacterial strains.

	MIC (µg mL^−1^) *		
Bacterial Strains	Methanol Extract	Water Extract	Ciprofloxacin
*Escherichia coli* (ATCC 10536)	396.85 (250–500)	629.96 (500–1000)	<0.12
*Pseudomonas aeruginosa* (ATCC 15442)	629.96 (500–1000)	314.98 (250–500)	1.23 (1.95–0.98)
*Salmonella typhimurium* (clinical isolate)	793.70 (500–1000)	793.70 (500–1000)	0.40 (0.25–0.5)
*Bacillus cereus* (ATCC 12826)	198.42 (125–250)	157.49 (125–250)	<0.12
*Bacillus subtilis* (environmental isolate)	314.98 (250–500)	793.70 (500–1000)	0.01 (0.125–0.062)
*Staphylococcus aureus* (ATCC 6538)	198.42 (125–250)	396.85 (250–500)	0.62 (0.98–0.49)

* MIC values are reported as geometric means of three independent replicates (*n* = 3); MIC range concentrations are reported within brachets.

**Table 7:** Gallic acid, catechin and epicatechin level (µg/g dry extract) in methanol and water extracts of *B. speciosa.*

Compounds	Methanol Extract	Water Extract
Gallic acid	7228.36 ± 650.55	870.28 ± 36.81
Catechin	20.84 ± 2.51	n.d.
Epicatechin	188.72 ± 11.32	142.71 ± 7.75

n.d.—not determined.

## Supplementary Materials

The following are available online at https://www.mdpi.com/2076-3921/9/2/128/s1. Supplementary Materials and Methods; CFF-strains.

## References

[1] Kebebew M., Dadi K., Mohammed E. (2017). Diversity, Knowledge and Use of Traditional Medicinal Plants in Guduru District, Horo Guduru Wollega Zone, Oromia Region of Ethiopia. J. Med. Plants Stud..

[2] Ahmad Khan M.S., Ahmad I., Ahmad Khan M.S., Ahmad I., Chattopadhyay D. (2019). Chapter 1-Herbal Medicine: Current Trends and Future Prospects. New Look to Phytomedicine.

[3] Feng S., Cheng S., Yuan Z., Leitch M., Xu C.C. (2013). Valorization of bark for chemicals and materials: A review. Renew. Sustain. Energy Rev..

[4] Tanase C., Coșarcă S., Muntean D.-L. (2019). A Critical Review of Phenolic Compounds Extracted from the Bark of Woody Vascular Plants and Their Potential Biological Activity. Molecules.

[5] Ferrante C., Recinella L., Ronci M., Orlando G., Di Simone S., Brunetti L., Chiavaroli A., Leone S., Politi M., Tirillini B. (2019). Protective effects induced by alcoholic Phlomis fruticosa and Phlomis herba-venti extracts in isolated rat colon: Focus on antioxidant, anti-inflammatory, and antimicrobial activities in vitro. Phytother. Res..

[6] Orlando G., Ferrante C., Zengin G., Sinan K.I., Bene K., Diuzheva A., Jekő J., Cziáky Z., Di Simone S., Recinella L. (2019). Qualitative Chemical Characterization and Multidirectional Biological Investigation of Leaves and Bark Extracts of Anogeissus leiocarpus (DC.) Guill. & Perr (Combretaceae). Antioxidants.

[7] Tanase C., Mocan A., Coșarcă S., Gavan A., Nicolescu A., Gheldiu A.-M., Vodnar D.C., Muntean D.-L., Crișan O. (2019). Biological and chemical insights of beech (*Fagus sylvatica* L.) bark: A source of bioactive compounds with functional properties. Antioxidants.

[8] Maroyi A. (2017). Ethnopharmacology and therapeutic value of Bridelia micrantha (Hochst.) Baill. In tropical Africa: A comprehensive review. Molecules.

[9] Ngueyem T.A., Brusotti G., Caccialanza G., Finzi P.V. (2009). The genus Bridelia: A phytochemical and ethnopharmacological review. J. Ethnopharmacol..

[10] Afolabi O.B., Oloyede O.I., Agunbiade S.O. (2018). Inhibitory potentials of phenolic-rich extracts from Bridelia ferruginea on two key carbohydrate-metabolizing enzymes and Fe2+-induced pancreatic oxidative stress. J. Integr. Med..

[11] Corallo A., Foungbé S., Davy M., Cohen Y. (1997). Cardiovascular pharmacology of aqueous extract of the leaves of Bridelia atroviridis Muell. Arg. (Euphorbiaceae) in the rat. J. Ethnopharmacol..

[12] Olajide O.A., Makinde J.M., Okpako D.T., Awe S.O. (2000). Studies on the anti-inflammatory and related pharmacological properties of the aqueous extract of Bridelia ferruginea stem bark. J. Ethnopharmacol..

[13] Ramesh N., Viswanathan M.B., Saraswathy A., Brindha P., Balakrishna K., Lakshmanaperumalsamy P. (2001). Antibacterial activity of luteoforol from Bridelia crenulata. Fitoterapia.

[14] Sokeng S., Rokeya B., Mostafa M., Nahar N., Mosihuzzaman M., Ali L., Kamtchouing P. (2005). Antihyperglycemic effect of Bridelia ndellensis ethanol extract and fractions in streptozotocin-induced diabetic rats. Afr. J. Tradit. Complement. Altern. Med..

[15] Tatiya A.U., Saluja A.K., Kalaskar M.G., Surana S.J., Patil P.H. (2017). Evaluation of analgesic and anti-inflammatory activity of Bridelia retusa (Spreng) bark. J. Tradit. Complement. Med..

[16] Cimanga K., Ying L., De Bruyne T., Apers S., Cos P., Hermans N., Bakana P., Tona L., Kambu K., Kalenda D. (2001). Radical scavenging and xanthine oxidase inhibitory activity of phenolic compounds from Bridelia ferruginea stem bark. J. Pharm. Pharmacol..

[17] Jayasinghe L., Kumarihamy B.M., Jayarathna K.H., Udishani N.W., Bandara B.M., Hara N., Fujimoto Y. (2003). Antifungal constituents of the stem bark of Bridelia retusa. Phytochemistry.

[18] Pegel K.H., Rogers C.B. (1968). Constituents of Bridelia micrantha. Phytochemistry.

[19] Uysal S., Aktumsek A. (2015). A phytochemical study on Potentilla anatolica: An endemic Turkish plant. Ind. Crop. Prod..

[20] Vladimir-Knezevic S., Blazekovic B., Stefan M.B., Alegro A., Koszegi T., Petrik J. (2011). Antioxidant activities and polyphenolic contents of three selected Micromeria species from Croatia. Molecules.

[21] Zengin G., Aktumsek A. (2014). Investigation of antioxidant potentials of solvent extracts from different anatomical parts of Asphodeline anatolica E. Tuzlaci: An endemic plant to Turkey. Afr. J. Tradit. Complement. Altern. Med..

[22] Di Giacomo V., Ferrante C., Ronci M., Cataldi A., Di Valerio V., Rapino M., Recinella L., Chiavaroli A., Leone S., Vladimir-Knežević S. (2019). Multiple pharmacological and toxicological investigations on Tanacetum parthenium and Salix alba extracts: Focus on potential application as anti-migraine agents. Food Chem. Toxicol..

[23] Grochowski D.M., Uysal S., Aktumsek A., Granica S., Zengin G., Ceylan R., Locatelli M., Tomczyk M. (2017). In vitro enzyme inhibitory properties, antioxidant activities, and phytochemical profile of Potentilla thuringiaca. Phytochem. Lett..

[24] Zengin G., Ferrante C., Gnapi D.E., Sinan K.I., Orlando G., Recinella L., Diuzheva A., Jekő J., Cziáky Z., Chiavaroli A. (2019). Comprehensive approaches on the chemical constituents and pharmacological properties of flowers and leaves of American basil (*Ocimum americanum* L.). Food Res. Int..

[25] Zengin G., Ferrante C., Orlando G., Zheleva-Dimitrova D., Gevrenova R., Recinella L., Chiavaroli A., Leone S., Brunetti L., Aumeeruddy M.Z. (2019). Chemical profiling and pharmaco-toxicological activity of Origanum sipyleum extracts: Exploring for novel sources for potential therapeutic agents. J. Food Biochem..

[26] Veschi S., De Lellis L., Florio R., Lanuti P., Massucci A., Tinari N., De Tursi M., di Sebastiano P., Marchisio M., Natoli C. (2018). Effects of repurposed drug candidates nitroxoline and nelfinavir as single agents or in combination with erlotinib in pancreatic cancer cells. J. Exp. Clin. Cancer Res..

[27] Ferrante C., Recinella L., Locatelli M., Guglielmi P., Secci D., Leporini L., Chiavaroli A., Leone S., Martinotti S., Brunetti L. (2017). Protective effects induced by microwave-assisted aqueous Harpagophytum extract on rat cortex synaptosomes challenged with amyloid β-peptide. Phytother. Res..

[28] Gan R.-Y., Chan C.-L., Yang Q.-Q., Li H.-B., Zhang D., Ge Y.-Y., Gunaratne A., Ge J., Corke H., Feng H., Nemzer B., DeVries J.W. (2019). 9-Bioactive compounds and beneficial functions of sprouted grains. Sprouted Grains.

[29] Panche A.N., Diwan A.D., Chandra S.R. (2016). Flavonoids: An overview. J. Nutr. Sci..

[30] Prasain J.K., Barnes S., Michael Wyss J., Watson R.R., Preedy V.R., Zibadi S. (2018). Chapter 24—Analyzing Ingredients in Dietary Supplements and Their Metabolites. Polyphenols: Mechanisms of Action in Human Health and Disease.

[31] Chandrasekara A., Melton L., Shahidi F., Varelis P. (2019). Phenolic Acids. Encyclopedia of Food Chemistry.

[32] Menon R., Gonzalez T., Ferruzzi M., Jackson E., Winderl D., Watson J., Henry J. (2016). Chapter One-Oats—From Farm to Fork. Advances in Food and Nutrition Research.

[33] Nabeelah Bibi S., Fawzi M.M., Gokhan Z., Rajesh J., Nadeem N., Kannan R.R., RDDG A., Pandian S.K. (2019). Ethnopharmacology, Phytochemistry, and Global Distribution of Mangroves—A Comprehensive Review. Mar. Drugs.

[34] Mahomoodally M.F., Mollica A., Stefanucci A., Aumeeruddy M.Z., Poorneeka R., Zengin G. (2018). Volatile components, pharmacological profile, and computational studies of essential oil from Aegle marmelos (Bael) leaves: A functional approach. Ind. Crop. Prod..

[35] McDonald S., Prenzler P.D., Antolovich M., Robards K. (2001). Phenolic content and antioxidant activity of olive extracts. Food Chem..

[36] Reddy N.S., Navanesan S., Sinniah S.K., Wahab N.A., Sim K.S. (2012). Phenolic content, antioxidant effect and cytotoxic activity of Leea indica leaves. BMC Complement. Altern. Med..

[37] Lobo V., Patil A., Phatak A., Chandra N. (2010). Free radicals, antioxidants and functional foods: Impact on human health. Pharmacol. Rev..

[38] Baloglu M.C., Llorent-Martínez E.J., Aumeeruddy M.Z., Mahomoodally M.F., Altunoglu Y.C., Ustaoglu B., Ocal M., Gürel S., Bene K., Sinan K.I. (2019). Multidirectional insights on Chrysophyllum perpulchrum leaves and stem bark extracts: HPLC-ESI-MSn profiles, antioxidant, enzyme inhibitory, antimicrobial and cytotoxic properties. Ind. Crop. Prod..

[39] Mahomoodally M.F., Picot-Allain C., Hosenally M., Ugurlu A., Mollica A., Stefanucci A., Llorent-Martínez E.J., Baloglu M.C., Zengin G. (2019). Multi-targeted potential of Pittosporum senacia Putt.: HPLC-ESI-MSn analysis, in silico docking, DNA protection, antimicrobial, enzyme inhibition, anti-cancer and apoptotic activity. Comput. Biol. Chem..

[40] Kıvrak E.G., Yurt K.K., Kaplan A.A., Alkan I., Altun G. (2017). Effects of electromagnetic fields exposure on the antioxidant defense system. J. Microsc. Ultrastruct..

[41] Bhagavan N.V., Ha C.-E., Bhagavan N.V., Ha C.-E. (2011). Chapter 6-Enzymes and Enzyme Regulation. Essentials of Medical Biochemistry.

[42] Klimova B., Kuca K., Maresova P. (2018). Global View on Alzheimer’s Disease and Diabetes Mellitus: Threats, Risks and Treatment Alzheimer’s Disease and Diabetes Mellitus. Curr. Alzheimer Res..

[43] Lee H.J., Seo H.I., Cha H.Y., Yang Y.J., Kwon S.H., Yang S.J. (2018). Diabetes and Alzheimer’s Disease: Mechanisms and Nutritional Aspects. Clin. Nutr. Res..

[44] Femminella G.D., Frangou E., Love S.B., Busza G., Holmes C., Ritchie C., Lawrence R., McFarlane B., Tadros G., Ridha B.H. (2019). Evaluating the effects of the novel GLP-1 analogue liraglutide in Alzheimer’s disease: Study protocol for a randomised controlled trial (ELAD study). Trials.

[45] Picot M.C., Zengin G., Mollica A., Stefanucci A., Carradori S., Mahomoodally M. (2017). In vitro and in silico studies of mangiferin from Aphloia theiformis on key enzymes linked to diabetes type 2 and associated complications. Med. Chem..

[46] Fang L., Chen M., Liu Z., Fang X., Gou S., Chen L. (2016). Ferulic acid—Carbazole hybrid compounds: Combination of cholinesterase inhibition, antioxidant and neuroprotection as multifunctional anti-Alzheimer agents. Bioorgan. Med. Chem..

[47] Mo J., Yang H., Chen T., Li Q., Lin H., Feng F., Liu W., Qu W., Guo Q., Chi H. (2019). Design, synthesis, biological evaluation, and molecular modeling studies of quinoline-ferulic acid hybrids as cholinesterase inhibitors. Bioorgan. Chem..

[48] Li N., Lin Z., Chen W., Zheng Y., Ming Y., Zheng Z., Huang W., Chen L., Xiao J., Lin H. (2018). Corilagin from longan seed: Identification, quantification, and synergistic cytotoxicity on SKOv3ip and hey cells with ginsenoside Rh2 and 5-fluorouracil. Food Chem. Toxicol..

[49] Shi Z.-L., Liu Y.-D., Yuan Y.-Y., Song D., Qi M.-F., Yang X.-J., Wang P., Li X.-Y., Shang J.-H., Yang Z.-X. (2017). In Vitro and In Vivo Effects of Norathyriol and Mangiferin on α-Glucosidase. Biochem. Res. Int..

[50] Chu H., Duan Y., Lang S., Jiang L., Wang Y., Llorente C., Liu J., Mogavero S., Bosques-Padilla F., Abraldes J.G. (2019). The Candida albicans exotoxin Candidalysin promotes alcohol-associated liver disease. J. Hepatol..

[51] Khiewkamrop P., Phunsomboon P., Richert L., Pekthong D., Srisawang P. (2018). Epistructured catechins, EGCG and EC facilitate apoptosis induction through targeting de novo lipogenesis pathway in HepG2 cells. Cancer Cell Int..

[52] Abnosi M.H., Yari S. (2018). The toxic effect of gallic acid on biochemical factors, viability and proliferation of rat bone marrow mesenchymal stem cells was compensated by boric acid. J. Trace Elem. Med. Biol..

[53] Mole D., McFerran N., Collett G., O’Neill C., Diamond T., Garden O., Kylanpaa L., Repo H., Deitch E. (2008). Tryptophan catabolites in mesenteric lymph may contribute to pancreatitis-associated organ failure. Br. J. Surg. Inc. Eur. J. Surg. Swiss Surg..

[54] Nakagami Y., Saito H., Katsuki H. (1996). 3-Hydroxykynurenine toxicity on the rat striatum in vivo. Jpn. J. Pharmacol..

[55] Han X., Li B., Ye X., Mulatibieke T., Wu J., Dai J., Wu D., Ni J., Zhang R., Xue J. (2017). Dopamine D2 receptor signalling controls inflammation in acute pancreatitis via a PP2A-dependent Akt/NF-κB signalling pathway. Br. J. Pharmacol..

[56] Zhou H., Tang L., Yang Y., Lin L., Dai J., Ge P., Ai Q., Jiang R., Zhang L. (2018). Dopamine alleviated acute liver injury induced by lipopolysaccharide/d-galactosamine in mice. Int. Immunopharmacol..

